# Effects of Exercise Combined with Undenatured Type II Collagen on Endurance Capacity, Antioxidant Status, Muscle Lipogenic Genes and E3 Ubiquitin Ligases in Rats

**DOI:** 10.3390/ani11030851

**Published:** 2021-03-17

**Authors:** Cemal Orhan, Emre Sahin, Besir Er, Mehmet Tuzcu, Andrey P. Lopes, Nurhan Sahin, Vijaya Juturu, Kazim Sahin

**Affiliations:** 1 Animal Nutrition Department, Veterinary Faculty, University of Firat, 23119 Elazig, Turkey; corhan@firat.edu.tr (C.O.); esahin@bingol.edu.tr (E.S.); nsahin@firat.edu.tr (N.S.); 2 Division of Biology, Science Faculty, Firat University, 23119 Elazig, Turkey; beshir.er@hotmail.com (B.E.); mtuzcu@firat.edu.tr (M.T.); 3 Department of Development & Innovation, Lonza, Rio de Janeiro 22793, Brazil; andrey.lopes@lonza.com; 4 Department of Research & Development, Lonza, Morristown, NJ 07960, USA; vijaya.juturu@lonza.com

**Keywords:** exercise, endurance, undenatured type II collagen, anti-inflammatory, antioxidants, immune response

## Abstract

**Simple Summary:**

Undenatured type II collagen (UCII), a collagen product that modulates the immune system by oral tolerance, has become a novel alternative agent to support skeletal system health. The current study explored the impact of UCII on endurance capacity, oxidative stress, inflammation, and antioxidant defense markers in exercised rats. UCII supplementation decreased serum lactate, malondialdehyde, inflammatory marker levels (TNF-α) and improved antioxidant status and lipid metabolism in training rats.

**Abstract:**

The current study aimed to investigate the effect of exercise combined with undenatured type II collagen (UCII) administration on endurance capacity, lipid metabolism, inflammation, and antioxidant status in rats. Twenty-one male Wistar albino rats were divided into three groups as follows: (1) Sedentary control, (2) Exercise (E), (3) Exercise + UCII (4 mg/kg BW/day; E + UCII). The findings showed that the exhaustive running time in the UCII group was significantly prolonged compared to that of the non-supplemented group (*p* < 0.001). When compared to the control group, total serum cholesterol (TC, *p* < 0.05) and triglyceride (TG, *p* < 0.05) levels decreased, while creatinine kinase (CK) levels increased in the E group (*p* < 0.001). Serum creatinine kinase levels were reduced in the E + UCII group compared to the E group (*p* < 0.01). Serum lactate, myoglobin (*p* < 0.01), and osteocalcin levels (*p* < 0.01) increased significantly in exercised rats compared to sedentary control rats, while serum lactate (*p* < 0.01) and myoglobin (*p* < 0.0001) levels decreased in the E + UCII group compared to control. Additionally, UCII supplementation caused significant increases in antioxidant enzyme activities [SOD (*p* < 0.01) and GSH-Px (*p* < 0.05)] and decreases in malondialdehyde (MDA) and tumor necrosis factor (TNF-α) levels (*p* < 0.001). Muscle lipogenic protein (SREBP-1c, ACLY, LXR, and FAS) levels were lower in the E + UCII group than in other groups. In addition, UCII supplementation decreased muscle MAFbx, MuRF-1, myostatin and increased MyoD levels in exercised rats. Moreover, the E + UCII group had lower muscle inflammatory markers [TNF-α (*p* < 0.0001) and IL-1β (*p* < 0.01)] than the control group. These results suggest exercise combined with UCII (4 mg/kg BW/day) modulates lipid, muscle, and antioxidant status in rats.

## 1. Introduction

Exercise is essential for improving functional capacity, cognitive function and preventing chronic diseases [1]. Many studies have demonstrated the importance of regular exercise in stimulating numerous metabolic health benefits such as metabolic syndrome, hypertension, muscle metabolism, and the antioxidant system [2,3,4,5,6]. Regular exercise strengthens muscle and bone quality around the joint, helps control weight, combat fatigue, and improves life [7]. Hurley et al. [8] reported reduced pain with an inevitable decline of 6%, equivalent to a 1.25-point reduction (improvement) in a review article of 9 clinical studies. In the same report, five exercise studies on health-related quality of life using the 36-item Short Form reported substantial social function benefits with an absolute percent of 7.9% [8]. Regular exercise can stimulate glycosaminoglycan content in cartilage and prevent cartilage damage, including loss of extracellular matrix, inflammation development, and osteophyte formation [9,10].

Regular exercise has a vital role in regulating metabolic function, such as carbohydrate and lipid metabolism [11]. Metabolism disorders caused by insufficient exercise or unbalanced nutrition can induce obesity, diabetes [12], lipogenic genes [sterol regulatory element-binding protein 1 (SREBP-1), liver X receptors (LXR), ATP-citrate lyase (ACL), and fatty acid synthase (FAS)] defects [13], weakness of antioxidant status and inflammation [14]. On the other hand, few studies have found that endurance training increases energy expenses and can trigger lipolytic hormones to facilitate post-exercise energy expenditure [15].

Muscle protein synthesis and degradation are regulated by several signaling pathways, such as the mammalian target of rapamycin (mTOR) and the ubiquitin-proteasome pathways [16]. E3 ubiquitin ligases, including muscle ring finger 1 (MuRF1) and muscle atrophy F-box protein (MAFbx), targets muscle proteins as substrate. During cachexia, MuRF1 and MAFbx are expressed explicitly in atrophying skeletal muscle and mediate muscle protein degradation [17]. Lokireddy et al. [18] demonstrated MAFbx/atrogin-1 activates the breakdown of the myogenic transcription factors MyoD, while Cohen et al. [19] suggested MuRF1 is mainly involved in the degradation of myofibrillar proteins such as myosin heavy chain protein and myosin-binding protein C. Function loses in joints may reduce muscle strength, subsequently muscle mass. Depending on inflammatory inductors, tibialis anterior and quadriceps muscles probably undergo atrophy during knee osteoarthritis (OA), associated with increased MuRF1 expression [20]. Because joint health is closely related to muscle activity and mass [21,22], muscle integrity and joint health can affect each other and provides better endurance capacity in exercise.

The undenatured form of type II collagen (UCII) is safe, non-toxic, and has high antigenic specificity. It is derived from chicken sternum cartilage and is a powdered, glycosylated, and shelf-resistant component [23]. UCII modulates the immune system by oral tolerance, has become a novel alternative agent to support skeletal muscle system health over the past two decades [23,24]. Small amounts of UCII taken orally interact with gut-associated lymphoid tissue, where the naive T cells (Th0) transform into T regulatory cells (Treg) targeting the type II collagen [25]. When these specific Treg cells encounter type II collagen, recognized as an antigen by the immune system, they prevent autoimmune reactions by reducing killer T cell attacks on joint cartilage and stimulates anti-inflammatory cytokine production [25]. Subjects receiving UCII supplementation presented a vital improvement in knee extension compared to placebo [26]. One study reported that UCII increased daily activities, improved quality of life, and no side effects [27]. In a multicenter, randomized study comparing the efficacy and tolerability of UCII and glucosamine hydrochloride plus chondroitin sulfate for six months, the WOMAC score was improved with UCII compared with GC [28]. Previous studies have shown that small UCII doses modulate joint health in arthritis [27]. Tong et al. [29] demonstrated that ingestion of microgram amounts of UCII reduced inflammatory cytokine levels and possibly served to reduce both the incidence and severity of arthritis.

Although there are several inconsistent studies regarding the effects of exercise on inflammation and antioxidant enzyme activities, regular exercise can enhance functional capacity by balancing oxidation processes through increasing resistance against oxidative stress and accelerating recovery from its harmful effects [1,4,5,30]. Several studies have shown that exercise can regulate the mitochondrial antioxidant enzymes and the activity of DNA repair enzymes [31] by reducing the accumulation of lipid peroxidation markers [32] such as MDA in skeletal muscle cells [33]. On the other hand, Yan et al. [34] reported that UCII, a nutritional supplement, improves the antioxidant capacity of the body by increasing SOD activity and decreasing MDA content. They also stated that UCII could alleviate inflammation by regulating inflammatory cytokine levels. Many dietary supplements are widely used to improve performance and reduce muscle fatigue and/or possible damage during physical exercises [5,6,33]. Although there are reports about the positive effects of treadmill exercise on inflammation and antioxidant enzymes, no studies investigate the effects of exercise combined with UCII on endurance capacity, lipid metabolism, oxidative stress, and inflammation markers, lipogenic proteins, and E3 ubiquitin ligases. Hence, we investigated the effects of exercise combined with UCII supplementation on performance, inflammation including IL-1β, TNF-α, oxidative stress, and lipogenic proteins (SREBP-1c, ACLY, LXRs, FAS), and E3 ubiquitin ligases (MAFbx, MuRF-1) in regularly exercised rats.

## 2. Materials and Methods

### 2.1. Animals

Twenty-one male Wistar Albino rats (8 weeks old, 180 ± 20 g) were obtained from the Firat University (Elazig, Turkey) and housed in cages at 22 ± 2 °C and controlled lighting (12 h light and 12 h dark). The Firat University of Animal Ethical Committee, Elazig, Turkey (2019/139–206) approved all the study processes.

### 2.2. Experimental Design

Rats were divided into three groups as follows: (i) Sedentary control (*n* = 7), (ii) Exercise (E, *n* = 7), (iii) Exercise+ UCII [(E + UCII, *n* = 7); 4 mg/kg BW/day)]. UC-II^®^ formulation (lot number#1808021) as the powder was provided by Lonza Consumer Health Inc., Morristown, NJ, USA. UCII and physiological saline as a placebo were given daily by gavage before exercise during the trial period (8 weeks). According to the FDA, the animal dose was calculated by converting the human equivalent dose (HED) [35].

### 2.3. Exercise Procedure

The exercise was done on the treadmill (Commat Limited, Ankara, Turkey), which comprises a motivation grid at its rear end that provides an electric shock if the animal places in our previous studies [33]. Rats in exercise ran on the treadmill 25 m/min, 45 min/day, and five days per week for eight weeks [36]. This model offers adaptations to the cardiovascular system, comprising the heart representative’s physiological remodeling with improved O_2_ intake, enhancement of cardiac contractile function, and calcium utilization [36,37]. Exhaustion time and average distance run were noted at the end of each training session.

### 2.4. Sample Collection

After decapitation with cervical dislocation under anesthesia, blood and gastrocnemius muscle were taken. Serum samples were taken to biochemical gel tubes after centrifugation. The muscle samples were quickly removed and stored at −80 °C for further analysis. Tissue was homogenized within 10 min in 10 volumes of cold Tris 10 mM (pH 7.4). The homogenates were then centrifuged to give the low-speed supernatant fraction used for analysis.

### 2.5. Biochemical Analysis

Serum samples were analyzed for serum glucose, lipid profile, liver enzymes (AST, ALT), blood urea-N, and creatinine levels by the biochemical analyzer (Samsung Electronics Co., Suwon, Korea). Serum lactate (Cayman Chemical Co., Ann Arbor, MI, USA), myoglobin, and osteocalcin concentrations (MyBioSource, San Diego, CA, USA) were measured by ELISA (Elx-800, Bio-Tek Instruments Inc., Winooski, VT, USA) according to the manufacturer’s instructions. The intra- and interassay coefficients of variation for lactate, myoglobin, and osteocalcin kits were <15%. Serum cartilage oligomeric matrix protein (COMP), interleukin 1β, (IL-1β), IL-6, and tumor necrosis factor (TNF-α) levels were also analyzed with ELISA kits (MyBioSource, San Diego, CA, USA) according to the manufacturer’s instructions.

The malondialdehyde (MDA) level in samples was detected by HPLD (Shimadzu, Tokyo, Japan) using a UV-vis SPD-10 AVP detector and C18 ODS-3, 5 µm, 4.6 mm × 250 mm column. Antioxidant enzymes (SOD, CAT, GSHPx) were assessed by commercially available kits (Cayman Chemical, Ann Arbor, MI, USA) according to the manufacturer’s process.

### 2.6. Western Blot Method

Muscle SREBP-1c, ACLY, LXRs, FAS, MAFbx, MuRF-1, MyoD, Myostatin, IL-1β, TNF-α, and NCAM levels were determined using the Western blot technique [27]. The muscle homogenates were prepared in ice-cold lysis buffer. SDS-PAGE sample buffer containing 2% β-mercaptoethanol was added to the supernatant. Twenty micrograms of protein were electrophoresed and then transferred into nitrocellulose membranes (Schleicher and Schuell Inc., Keene, NH, USA). Nitrocellulose blots blocked with 1% bovine serum albumin in PBS for one hour prior to administration of the primary antibodies (SREBP-1c, ACLY, LXRs, FAS, MAFbx, MuRF-1, MyoD, Myostatin, TNF-α, IL-1β, and NCAM) (Abcam, Cambridge, UK) that were diluted (1:1000) in the same buffer containing 0.05% Tween-20. Protein loading was checked using an antibody against α-actin (A5316; Sigma Aldrich, St. Louis, MO, USA). Bands were analyzed densitometrically using an image analysis system (Image J; National Institute of Health, Bethesda, MA, USA).

### 2.7. Statistical Analysis

Data were noted as mean ± SE. The sample size is based on a power of 85% to achieve a *p*-value of 0.05. Seven animals were tested to get the significance of the treatments per group. Analyses were done with the SPSS software program (IBM SPSS, Version 22.0; Chicago, IL, USA). Significance was detected with a one-way ANOVA followed by a post-hoc Tukey test and determined as significant for probability values less than *p* < 0.05.

## 3. Results

### 3.1. Performance and Serum Analyses

**Figure 1:**
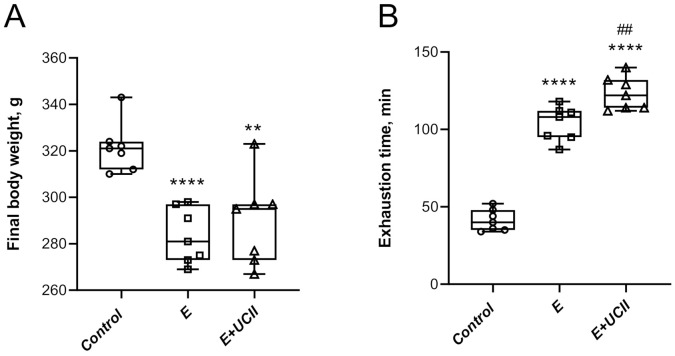
The effect of UCII on body weight (**A**) and exhaustion time (**B**) in exercised rats. Box and Whisker plots show median, min, and max values. ANOVA and Tukey’s post-hoc test were used to compare the results among different treatment groups. Statistical Scheme ** *p* < 0.01; **** *p* < 0.0001 compared to the control group and ^##^ *p* < 0.01 compared to the exercise group. UCII, Undenatured type II collagen. Control (Circle): no exercise and no UCII, E (Square): exercised rats, E + UCII (triangle), exercised rats receiving 4 mg/kg BW/day UCII.

Significant body weight changes were observed between the E group and the sedentary control group (*p* < 0.0001 for control vs. E; *p* < 0.01 for control vs. E + UCII; Figure 1A). Exhaustion time increased in the exercise and E + UCII groups compared to the control group (*p* < 0.0001; Figure 1B). Additionally, training combined with UCII increased the exhaustion time by 18.7% in exercised rats (*p* < 0.01). Moreover, while there was no difference between E + UCII and control groups, significant decreases were observed in serum cholesterol (4.0%) and triglyceride (6.0%) in group E compared to the control group (*p* < 0.05). Serum creatinine kinase concentration was higher in the E (*p* < 0.0001) and E + UCII groups compared to the control group (*p* < 0.01). There was no statistical change in liver and kidney function tests for all groups (*p* > 0.05; Table 1).

### 3.2. Inflammatory and Cartilage Markers

**Figure 2:**
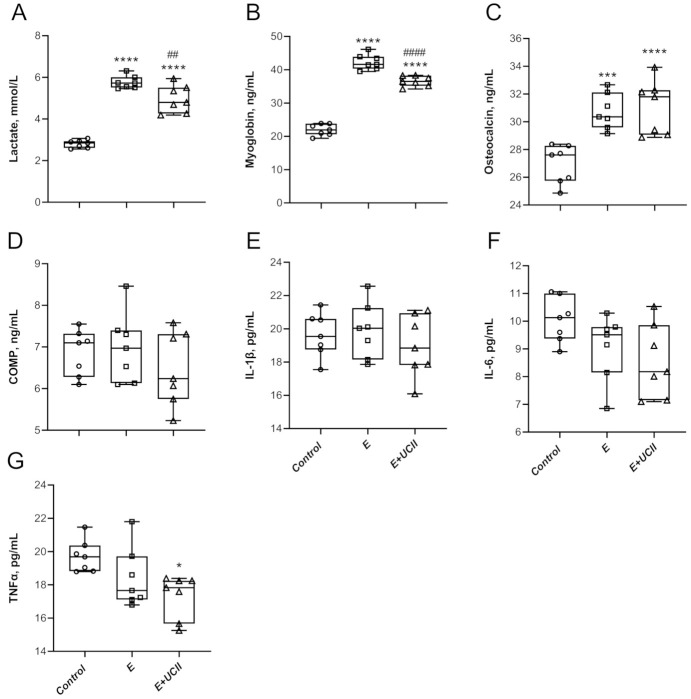
The effect of UCII on the serum lactate (**A**), myoglobin (**B**), osteocalcin (**C**), COMP (**D**), IL-1β (**E**), IL-6 (**F**), and TNFα (**G**) levels in exercised rats. Box and Whisker plots show median, min, and max values. ANOVA and Tukey’s post-hoc test were used to compare the results among different treatment groups. Statistical significance between groups is shown by: * *p* < 0.05, *** *p* < 0.001, **** *p* < 0.0001 compared as control group and, ^##^ *p* < 0.01, ^####^ *p* < 0.01 com-pared as exercise Group. UCII, Undenatured type II collagen; COMP, cartilage oligomeric matrix protein; IL-1β, interleukin-1β, IL-6, interleukin-6; TNFα, tumor necrosis factor-alpha. Control (Circle): no exercise and no UCII, E (Square): exercised rats, E + UCII (triangle), exercised rats receiving 4 mg/kg BW/day UCII.

Concentrations of lactate in serum increased by 2.1 and 1.8 fold in the E group and E + UCII group compared to the control group (*p* < 0.0001 for both; Figure 2A). It was lower by 13.8 in the E + UCII group than the E group (*p* < 0.01). Similarly, serum myoglobin concentrations in the E group and E + UCII group were 1.9 and 1.7 folds higher than in the control group. However, myoglobin concentrations in the E + UCII group were 13.6% lower than in the E group (*p* < 0.001; Figure 2B). Serum osteocalcin levels increased by 14.2% and 15.4% in the E (*p* < 0.0001) and E + UCII (*p* < 0.001) groups compared to the control group (Figure 2C), while osteocalcin levels did not change between E and E + UCII groups (*p* > 0.05). Serum COMP, IL-1β, and IL-6 concentrations were similar among groups (*p* > 0.05; Figure 2D–F). While there was no difference between the control group and E group in terms of serum TNF-α concentration (*p* > 0.05), it was 12.2% lower in the E + UCII group than in the control group (*p* < 0.05; Figure 2G).

### 3.3. Oxidative Stress and Antioxidant Properties

**Figure 3:**
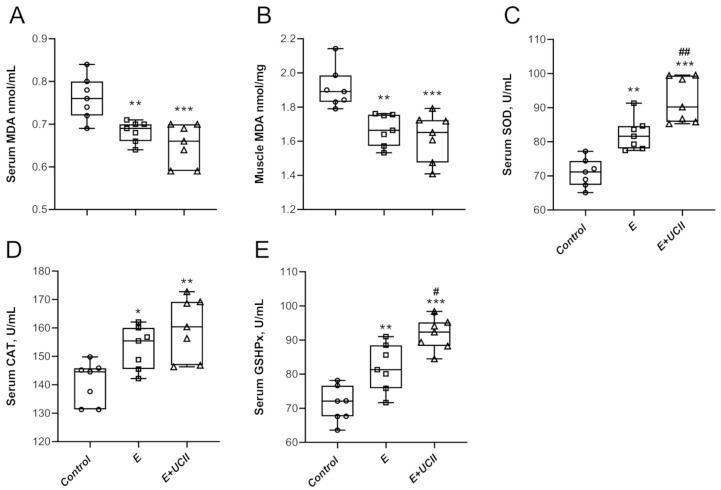
The effects of UCII on the serum MDA (**A**), muscle MDA (**B**), serum SOD (**C**), CAT (**D**), and GSHPx (**E**) levels in exercised rats. Box and Whisker plots show median, min, and max values. ANOVA and Tukey’s post-hoc test were used to compare the results among different treatment groups. Statistical significance between groups is shown by: * *p* < 0.05, ** *p* < 0.01, *** *p* < 0.001 compared as control group and ^#^ *p* < 0.05, ^##^ *p* < 0.01 compared as exercise Group. UCII, Undenatured type II collagen; MDA, malondialdehyde; SOD, superoxide dismutase, CAT, catalase; GSHPx, glutathione peroxidase, Control (Circle): no exercise and no UCII, E (Square): exercised rats, E + UCII (triangle), exercised rats receiving 4 mg/kg BW/day UCII.

A significant decrease in serum and muscle MDA levels of 10.3% and 12.7% was observed in group E compared to control (Figure 3A,B; *p* < 0.01 for both). Besides, serum and muscle MDA levels were 14.3% and 15.0% lower in the E + UCII group than the control group (*p* < 0.001 for both). Serum SOD, CAT, and GSHPx (Figure 3C–E) activities improved in the E and E + UCII groups compared to the control group. Serum SOD and GSHPx levels increased by 12.1% and 11.9% in the E + UCII group compared to the E group (*p* < 0.01 and *p* < 0.05).

### 3.4. Muscle Proteins and Inflammatory Cytokines

**Figure 4:**
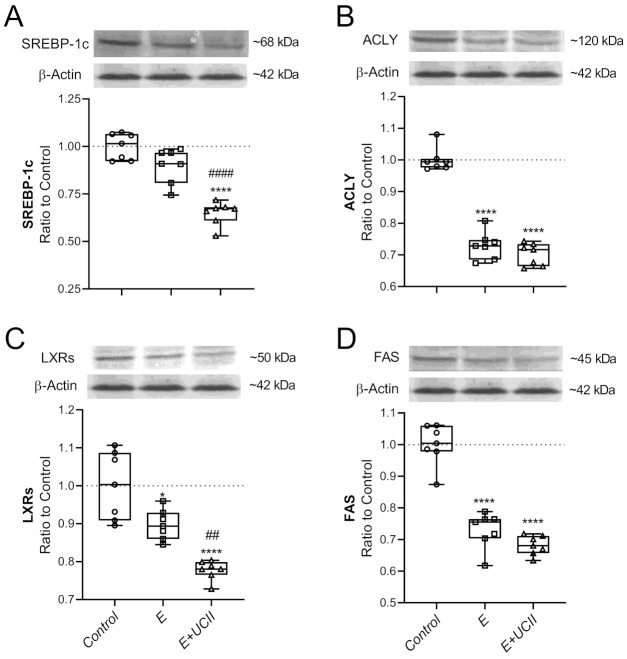
The effect of UCII on the muscle SREBP-1c (**A**), ACLY (**B**), LXRs (**C**), and FAS (**D**) levels in exercised rats. The densitometric analysis of the relative intensity according to the control group of the western blot bands (Figure S1) was performed with β-actin normalization to ensure equal protein loading. Blots were repeated at least three times (*n*  =  3), and a representative blot is shown. Box and Whisker plots show median, min, and max values. Data are expressed as a ratio of the control set at 1.0. ANOVA and Tukey’s post-hoc test were used to compare the results among different treatment groups. Statistical significance between groups is shown by: * *p* < 0.05; **** *p* < 0.0001 compared as control group and, ^##^ *p* < 0.01; ^####^ *p* < 0.0001 compared as exercise group. E: Exercise; UCII, Undenatured type II collagen; SREBP-1c, Sterol regulatory element-binding protein 1c; ACLY, ATP citrate lyase; LXRs, Liver X receptors; FAS, Fatty acid synthase, Control (Circle): no exercise and no UCII, E (Square): exercised rats, E + UCII (triangle), exercised rats receiving 4 mg/kg BW/day UCII.

While exercise alone did not alter the muscle SREBP-1c level, with UCII supplementation, the muscle SREBP-1c was reduced by 35.1% compared to the control (*p* < 0.0001; Figure 4A). Muscle SREBP-1c in the E + UCII group decreased by 27.8% compared to the E group (*p* < 0.0001). In the E + UCII and E groups, muscle ACLY levels reduced by 29.8% and 27.0% (*p* < 0.0001) and did not change at ACLY levels between the E and E + UCII groups (*p* > 0.05; Figure 4B).

LXRs level was lower in E and E + UCII groups than the control group (*p* < 0.05 and *p* < 0.0001; Figure 4C). Moreover, the LXRs level in the E + UCII group was lower than the E group (*p* < 0.01). The exercise alone and exercise combined with UCII supplementation decreased muscle FAS levels (*p* < 0.0001 for both; Figure 4D). However, the muscle FAS levels of E and E + UCII groups were similar (*p* > 0.05; Figure 4D).

**Figure 5:**
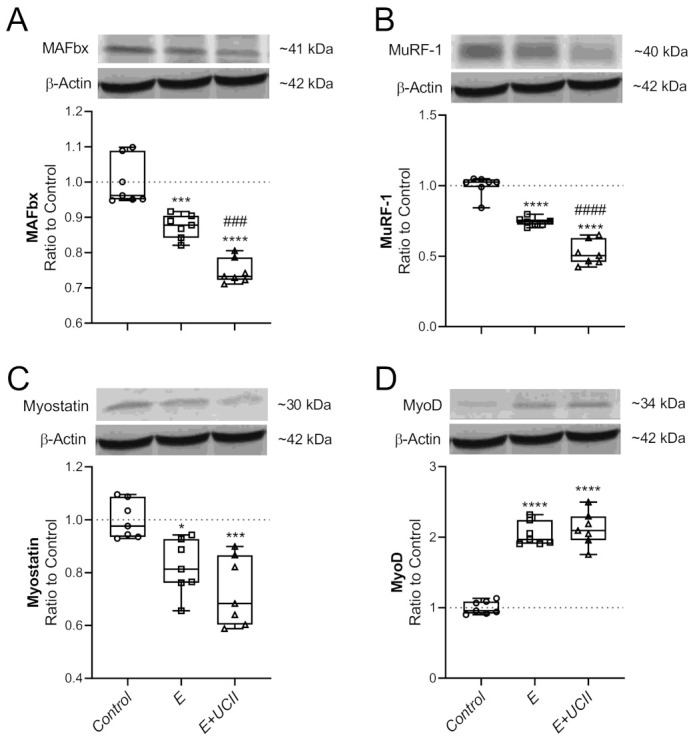
The effect of UCII on the muscle MAFbx (**A**), MuRF-1 (**B**), Myostatin (**C**), and MyoD (**D**) levels in exercised rats. The densitometric analysis of the relative intensity according to the control group of the western blot bands (Figure S2) was performed with β-actin normalization to ensure equal protein loading. Blots were repeated at least three times (*n*  =  3), and a representative blot is shown. Box and Whisker plots show median, min, and max values. Data are expressed as a ratio of the control set at 1.0. ANOVA and Tukey’s post-hoc test were used to compare the results among different treatment groups. Statistical significance between groups is shown by: * *p* < 0.05; *** *p* < 0.001; **** *p* < 0.0001 compared as control group and, ^###^ *p* < 0.001; ^####^ *p* < 0.0001 compared as exercise group. E: Exercise; UCII, Undenatured type II collagen; MAFbx, Muscle atrophy F-box; MuRF-1, Muscle RING-finger protein-1; MyoD, Myogenic differentiation factor, Control (Circle): no exercise and no UCII, E (Square): exercised rats, E + UCII (triangle), exercised rats receiving 4 mg/kg BW/day UCII.

Muscle MAFbx, MuRF-1, and Myostatin levels decreased in the E group compared to the control group (*p* < 0.001, *p* < 0.0001 *p* < 0.05, respectively; Figure 5A–C). Muscle MAFbx, MuRF-1, and Myostatin levels were lower in the E + UCII group compared to the control group (*p* < 0.0001, *p* < 0.0001 *p* < 0.001, respectively). However, muscle MAFbx and MuRF1 levels in the E + UCII group were lower than the E group (*p* < 0.001 and *p* < 0.0001). Muscle MyoD levels increased 2.0 and 2.1 fold in E and E + UCII groups compared to the control group (*p* < 0.0001 for both; Figure 5D).

**Figure 6:**
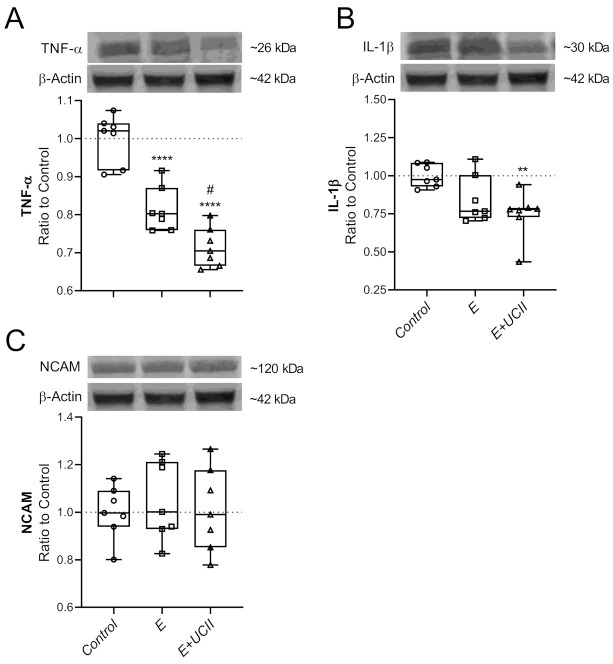
The effect of UCII on the muscle TNF-α (**A**), IL-1β (**B**), and NCAM (**C**) levels in exercised rats. The densitometric analysis of the relative intensity according to the control group of the western blot bands (Figure S3) was performed with β-actin normalization to ensure equal protein loading. Blots were repeated at least three times (*n*  =  3), and a representative blot is shown. Box and Whisker plots show median, min, and max values. Data are expressed as a ratio of the control set at 1.0. ANOVA and Tukey’s post-hoc test were used to compare the results among different treatment groups. Statistical significance between groups is shown by: ** *p* < 0.01; **** *p* < 0.0001 compared to the control group and, ^#^ *p* < 0.05 compared to the exercise group. E: Exercise; UCII, Undenatured type II collagen; TNF-α, Tumor necrosis factor-α; IL-1β, Interleukin-1β; NCAM, Neural cell adhesion molecules, Control (Circle): no exercise and no UCII, E (Square): exercised rats, E + UCII (triangle), exercised rats receiving 4 mg/kg BW/day UCII.

Supplementing UCII to exercised rats resulted in a 28.6% reduction in TNF-α levels compared to control rats (*p* < 0.0001; Figure 6A), and similarly, the exercise group reduced TNF-α levels by 18.6 compared to the control groups (*p* < 0.0001). The decrease in muscle TNF levels was more in the E + UCII group than the E group (*p* < 0.05). While muscle IL-1β level did not change in the E group (*p* > 0.05), it decreased by 25.4% in the E + UCII group compared to the control group. Muscle NCAM level neither changed in the E + UCII group nor the E group compared to the control group (*p* > 0.05 for both; Figure 6C).

## 4. Discussion

This study investigated the efficacy and possible mechanisms of UCII supplementation associated with endurance capacity, lipid metabolism, cartilage markers, and modulation of cartilage markers and antioxidants, and reduced oxidative stress and reduced oxidative stress, regulated muscle lipogenic and E3 Ubiquitin ligases proteins, as well as inflammatory markers including IL-1β and TNF-α in exercised rats.

The mechanism of action of UCII would provide new insights on the development of natural anti-inflammatory properties and immune tolerance and new therapeutic approaches for joint health. The clinically authorized laboratory confirms that the active epitopes in UCII are resistant to digestion and preserve the undenatured 3D structure required to interact with Peyer’s patches and induce oral tolerance. Upon consumption, UCII is thought to be taken up by the Peyer’s patches to trigger immune cells. It transforms naive T cells into T regulatory (Treg) cells that specifically target type-2 collagen. Then, Treg cells migrate through the circulation. When they identify type-2 collagen in articular cartilage, these collagen-specific regulatory T cells slow down the inflammatory cytokines’ production by secreting anti-inflammatory mediators such as TGF-β, IL-4, and IL-10. This action supports anti-inflammatory and cartilage-protecting signaling pathways that prevent the immune system from damaging and erosion joint cartilage while promoting cartilage repair and regeneration. Next, UCII provides relief of joint damage symptoms, which is recognized as oral tolerance and modulating inflammatory pathways [24,25]. Importantly, muscular weakness is one of the most predisposing factors in the progression of OA symptoms. Inflammatory factors lead to presynaptic reflex inhibition and changes in the neuromuscular junctions; thus, muscle atrophy and muscle weakness may be observed after inflammatory status [20]. The current study indicates various modulating pathways to inhibit muscle damage and inflammation, as shown in Figure 1, Figure 2, Figure 3, Figure 4, Figure 5 and Figure 6.

IL-1β, considered a pronociceptive cytokine, maybe primarily antinociceptive in disease states characterized by thermal hyperalgesia [38]. It independently causes inflammatory responses and catabolic consequences and is combined with other mediators to the articular cartilage and other articular elements. IL-6, considered a cytokine, strongly stimulates immunity and improves inflammatory reaction [38]. According to Haseeb and Haqqi [39], the immune system’s participation in OA’s development and progression is critical in disease pathogenesis. Mueller and Tuan [40] reported that cytokines might disturb the catabolism and anabolism progressions, most vital in tissues subjected to high mechanical load, including human joints. Thus, there is a progressive articular cartilage degeneration, and this degeneration includes both inflammation and degradation and production processes, which together lead to a gradual loss of joint function and pain. For the first time, the present study results showed that UCII significantly reduced lactate, myoglobin levels and increased osteocalcin levels in exercised rats, suggesting that it could improve endurance capacity. However, there are no studies to compare the lactate, myoglobin, and osteocalcin levels obtained in the present study in exercised rats. Moreover, exercise combined with UCII supplementation did not affect serum COMP, IL-1β, IL-6 in rats. However, UCII supplementation significantly reduced TNFα levels in exercised rats, suggesting that it alleviates inflammation. A recent study conducted by Varney et al. [41] in dogs indicates that UCII supplementation reduces inflammation after exercise, thus supporting our findings. In addition, UCII supplementation for osteoarthritis and healthy subjects demonstrated that 40 mg once a day of UCII supplementation reported efficacy in supporting joint comfort, mobility, flexibility [26,28], and companion animal support joint health and function [23]. Bagi et al. [42] reported the weight-bearing preservation of injured leg capacity and the cancellous bone integrity, suggesting potential for preventing worsening of articular cartilage damage with UCII supplementation the OA rat model. Besides the anti-inflammatory function of UCII, it is prolonged exhaustion compared to the unsupplemented group depending on its joint protecting properties.

Exercise triggers reactive oxygen species formation that acts as significant mediators and cellular adaptations, modulation of antioxidant protection, and repair of oxidative damage [43]. The present study data are consistent with the earlier studies showing that regular training increases antioxidant capacity, possibly adaptation to increased oxidative stress [4,5,44]. In addition, we found that exercise combined with UCII supplementation increased the activities of antioxidant enzymes (SOD and GSHPx) in rats. It indicates UCII may help to improve the antioxidant capacity in exercised rats. Moreover, decreased muscle TNF-α and IL-1β levels support that UCII has anti-inflammation activity on muscle in exercised rats. Though there is no literature to compare the effects of UCII on antioxidant levels in rats that were exercised, similar findings were obtained in OA rats. For example, Yan et al. [34] reported that UCII increased serum SOD activity in OA rats.

SREBP-1c, directly stimulated by LXR, induces fatty acid and triglyceride production by upregulating some lipogenic genes, including FAS and ACLY [45]. In skeletal muscle, lipid utilization increases with exercise, and muscle SREBP-1c protein levels tend to elevate after training [46]. However, SREBP actions may be altered with the type of training and energy intake. For example, Jeong et al. [47] determined that 8-week low-fat diet and exercise program reduces muscle SREBP-1c and TNF-α levels in high-fat diet (HFD) fed C57BL/6J mice. Likewise, muscle LXR levels were reduced in HFD fed rats after regular exercise (4 weeks) [48]. Additionally, Smith et al. [49] observed a reduction of muscle SREBP-1c mRNA levels in men at the end of the six-month endurance exercise. In contrast, de Souza Cordeiro et al. [50] reported that gastrocnemius muscle SREBP-1c expression increased after 10-week aerobic exercise in rats. Long-term exercise and caloric restriction could also increase the SREBP-1c levels independently in gastrocnemius and soleus muscles in rats [51]. SREBP-1c, ACLY, FAS, and LXRs protein levels were reduced after exercise in the current study. UCII supplementation further lowered SREBP-1c and LXRs levels compared to the exercised group. Unfortunately, the effects of exercise on muscle lipid metabolism still unclear, and we could not reach any paper to discuss the impact of UCII on muscle lipid metabolism.

Muscle wasting is inevitably related to aging, and, lately, it has been revealed in patients with OA [52]. The ubiquitin ligase MAFbx shows a critical role in muscle loss through regulating MyoD degradation. MAFbx expression, enhanced by myostatin and inflammatory disorders like OA, in muscle inhibits MyoD, promoting muscle regeneration [53]. In mice, mRNA expression and protein levels of MAFbx decreased in muscle after exercise, whereas MyoD protein levels increased [54]. Moreover, MuRF1 plays a vital role in muscle remodeling and triggers muscle protein degradation via ubiquitination [55]. Following repeated resistance exercise, MuRF-1 and MAFbx levels could be inhibited in human muscle, promoting muscle regeneration [56]. Recently, Zeng et al. [57] proved that E3 ubiquitin ligases such as MuRF1 and Atrogin-1 decreased after different types of exercise in aged rats. Similarly, we found that exercise reduced MAFbx, MuRF-1, and myostatin levels in muscle, whereas MyoD levels increased. UCII treatment decreased MAFbx and MuRF-1 compared to the exercise group. Although there is inadequate data on the effect of UCII on muscle functions, these results showed that UCII might regulate muscle metabolism by regulating MAFbx, MuRF-1, and myostatin.

Our study has a limitation, primarily related to the lack of data in the UCII-receiving sedentary control group alone. We could not show whether the use of UCII in healthy non-exercised animals improves the measured parameters. In addition, how UCII regulates the expression of anti-inflammatory cytokines inhibits the synthesis of inflammatory cytokines also needs in healthy animals in further study. However, the primary goal of this study is to investigate the effects of UCII combined with exercise in rats. We suggest that training combined with UCII supplementation may improve lipid, muscle, and antioxidant status in the exercised rat model; however, more in-depth studies are needed to confirm it. On the other hand, animal studies are models based on indications and effects; they help human studies move on. Based on the results, human studies should be planned. These results are not to exaggerate to humans. Further human double-blind studies should be designed in exercise or sporting activities.

## 5. Conclusions

These results suggest UCII supplementation with exercise modulates lipid, muscle, and antioxidant status in the exercised rat model. The action of anti-inflammatory cytokines inhibits the synthesis of inflammatory cytokines in muscle. These results also contribute significantly to expanding the academic community’s knowledge of the increase in UCII benefits combined with physical exercise by modulating inflammatory markers and antioxidant status. Furthermore, clinical studies are needed to demonstrate the effects of exercise combined with UCII in large animals and humans.

## Figures and Tables

**Table 1:** The effects of UCII on serum parameters in exercised rats (*n* = 7).

Items	Control	Exercise (E)	E + UCII
Glucose, mg/dL	108.29 ± 3.19	102.14 ± 1.77	102.43 ± 1.65
TC, mg/dL	98.51 ± 1.66	92.06 ± 1.57 *	95.70 ± 1.48
Triglyceride, mg/dL	103.51 ± 1.17	96.58 ± 1.74 *	102.40 ± 1.82 ^#^
TP, g/dL	6.56 ± 0.19	6.59 ± 0.11	6.52 ± 0.17
Albumin, g/dL	3.43 ± 0.10	3.51 ± 0.09	3.53 ± 0.12
Globulin, g/dL	3.09 ± 0.12	3.29 ± 0.10	3.19 ± 0.10
ALT, U/L	97.29 ± 4.65	98.43 ± 5.42	95.43 ± 3.00
AST, U/L	118.43 ± 6.21	116.86 ± 6.28	114.43 ± 7.75
TBil, mg/dL	0.24 ± 0.01	0.24 ± 0.01	0.24 ± 0.01
CK, IU/L	125.80 ± 1.75	193.60 ± 2.86 ****	179.98 ± 2.81 ****^, ##^
Creatine, mg/dL	0.48 ± 0.03	0.47 ± 0.02	0.48 ± 0.04
BUN, mg/dL	20.47 ± 0.77	20.86 ± 0.23	19.44 ± 0.60

Data are expressed as mean± SE. ANOVA and Tukey’s post-hoc test were used to compare the results among different treatment groups. Statistical significance between groups is shown by: * *p* < 0.05; **** *p* < 0.0001 compared as control group and, ^#^ *p* < 0.05, ^##^ *p* < 0.01 compared as exercise Group. UCII, Undenatured type II collagen; TC, total cholesterol; TG, triglyceride; TP, total protein; ALT, alanine transaminase; AST, aspartate transaminase; TBil, total bilirubin; CK, creatinine kinase; BUN, blood urea nitrogen. Control: no exercise and no UCII, E: exercised rats, E + UCII, exercised rats receiving 4 mg/kg BW/day UCII.

## Data Availability

The data presented in this study are available within the article.

## Supplementary Materials

The following are available online at https://www.mdpi.com/2076-2615/11/3/851/s1, Figure S1. Full immunoblots related to Figure 4 (SREB-1C (A), ACLY (B), LXRs (C), FAS (D); β-Actin (E)); Figure S2. Full immunoblots related to Figure 5 (MAFbx (A), MuRF-1 (B), Myostatin (C), Myo D (D), and β-Actin (E)); Figure S3. Full immunoblots related to Figure 6 (TNF-α (A), IL1-β (B), NCAM (C) and β-Actin (D)).

## References

[1] Steele J., Fisher J., Skivington M., Dunn C., Arnold J., Tew G., Batterham A.M., Nunan D., O’Driscoll J.M., Mann S. (2017). A higher effort-based paradigm in physical activity and exercise for public health: Making the case for a greater emphasis on resistance training. BMC Public Health.

[2] Elokda A.S., Nielsen D.H. (2007). Effects of exercise training on the glutathione antioxidant system. Eur. J. Cardiovasc. Prev. Rehabil..

[3] Kemmler W., Von Stengel S., Engelke K., Kalender W.A. (2009). Exercise decreases the risk of metabolic syndrome in elderly females. Med. Sci. Sports Exerc..

[4] Rosety-Rodriguez M., Rosety I., Fornieles-Gonzalez G., Diaz-Ordonez A.J., Camacho A., Rosety M.A., Pardo A., Rosety M., Alvero R., Ordonez F.J. (2012). A 6-week training program increased muscle antioxidant system in elderly diabetic fatty rats. Med. Sci. Monit..

[5] Silva E.P., Borges L.S., Mendes-da-Silva C., Hirabara S.M., Lambertucci R.H. (2017). l-Arginine supplementation improves rats’ antioxidant system and exercise performance. Free Radic. Res..

[6] Sahin K., Orhan C., Tuzcu M., Sahin N., Erten F., Juturu V. (2018). Capsaicinoids improve consequences of physical activity. Toxicol. Rep..

[7] Musumeci G. (2015). Effects of exercise on physical limitations and fatigue in rheumatic diseases. World J. Orthop..

[8] Hurley M., Dickson K., Hallett R., Grant R., Hauari H., Walsh N., Stansfield C., Oliver S. (2018). Exercise interventions and patient beliefs for people with hip, knee or hip and knee osteoarthritis: A mixed methods review. Cochrane Database Syst. Rev..

[9] Iijima H., Aoyama T., Ito A., Tajino J., Yamaguchi S., Nagai M., Kiyan W., Zhang X., Kuroki H. (2016). Exercise intervention increases expression of bone morphogenetic proteins and prevents the progression of cartilage-subchondral bone lesions in a post-traumatic rat knee model. Osteoarthr. Cartil..

[10] Smith J.K. (2020). Exercise as an adjuvant to cartilage regeneration therapy. Int. J. Mol. Sci..

[11] Thyfault J.P., Bergouignan A. (2020). Exercise and metabolic health: Beyond skeletal muscle. Diabetologia.

[12] Hemmingsen B., Gimenez-Perez G., Mauricio D., Roque I.F.M., Metzendorf M.I., Richter B. (2017). Diet, physical activity or both for prevention or delay of type 2 diabetes mellitus and its associated complications in people at increased risk of developing type 2 diabetes mellitus. Cochrane Database Syst. Rev..

[13] Solinas G., Boren J., Dulloo A.G. (2015). De novo lipogenesis in metabolic homeostasis: More friend than foe?. Mol. Metab..

[14] Booth F.W., Roberts C.K., Laye M.J. (2012). Lack of exercise is a major cause of chronic diseases. Compr. Physiol..

[15] Irving B.A., Davis C.K., Brock D.W., Weltman J.Y., Swift D., Barrett E.J., Gaesser G.A., Weltman A. (2008). Effect of exercise training intensity on abdominal visceral fat and body composition. Med. Sci. Sports Exerc..

[16] Yoon M.S. (2017). mTOR as a Key Regulator in Maintaining Skeletal Muscle Mass. Front. Physiol..

[17] Glass D.J. (2010). Signaling pathways perturbing muscle mass. Curr. Opin. Clin. Nutr. Metab. Care.

[18] Lokireddy S., Wijesoma I.W., Sze S.K., McFarlane C., Kambadur R., Sharma M. (2012). Identification of atrogin-1-targeted proteins during the myostatin-induced skeletal muscle wasting. Am. J. Physiol. Cell Physiol..

[19] Cohen S., Brault J.J., Gygi S.P., Glass D.J., Valenzuela D.M., Gartner C., Latres E., Goldberg A.L. (2009). During muscle atrophy, thick, but not thin, filament components are degraded by MuRF1-dependent ubiquitylation. J. Cell Biol..

[20] Cunha J.E., Barbosa G.M., Castro P., Luiz B.L.F., Silva A.C.A., Russo T.L., Vasilceac F.A., Cunha T.M., Cunha F.Q., Salvini T.F. (2019). Knee osteoarthritis induces atrophy and neuromuscular junction remodeling in the quadriceps and tibialis anterior muscles of rats. Sci. Rep..

[21] Heiden T.L., Lloyd D.G., Ackland T.R. (2009). Knee joint kinematics, kinetics and muscle co-contraction in knee osteoarthritis patient gait. Clin. Biomech..

[22] Van der Esch M., Steultjens M., Knol D.L., Dinant H., Dekker J. (2006). Joint laxity and the relationship between muscle strength and functional ability in patients with osteoarthritis of the knee. Arthritis Rheum..

[23] Gencoglu H., Orhan C., Sahin E., Sahin K. (2020). Undenatured Type II Collagen (UC-II) in Joint Health and Disease: A Review on the Current Knowledge of Companion Animals. Animals.

[24] Bagchi D., Misner B., Bagchi M., Kothari S.C., Downs B.W., Fafard R.D., Preuss H.G. (2002). Effects of orally administered undenatured type II collagen against arthritic inflammatory diseases: A mechanistic exploration. Int. J. Clin. Pharmacol. Res..

[25] Park K.S., Park M.J., Cho M.L., Kwok S.K., Ju J.H., Ko H.J., Park S.H., Kim H.Y. (2009). Type II collagen oral tolerance; mechanism and role in collagen-induced arthritis and rheumatoid arthritis. Mod. Rheumatol..

[26] Lugo J.P., Saiyed Z.M., Lau F.C., Molina J.P., Pakdaman M.N., Shamie A.N., Udani J.K. (2013). Undenatured type II collagen (UC-II(R)) for joint support: A randomized, double-blind, placebo-controlled study in healthy volunteers. J. Int. Soc. Sports Nutr..

[27] Crowley D.C., Lau F.C., Sharma P., Evans M., Guthrie N., Bagchi M., Bagchi D., Dey D.K., Raychaudhuri S.P. (2009). Safety and efficacy of undenatured type II collagen in the treatment of osteoarthritis of the knee: A clinical trial. Int. J. Med. Sci..

[28] Lugo J.P., Saiyed Z.M., Lane N.E. (2016). Efficacy and tolerability of an undenatured type II collagen supplement in modulating knee osteoarthritis symptoms: A multicenter randomized, double-blind, placebo-controlled study. Nutr. J..

[29] Tong T., Zhao W., Wu Y.Q., Chang Y., Wang Q.T., Zhang L.L., Wei W. (2010). Chicken type II collagen induced immune balance of main subtype of helper T cells in mesenteric lymph node lymphocytes in rats with collagen-induced arthritis. Inflamm. Res..

[30] Radak Z., Chung H.Y., Koltai E., Taylor A.W., Goto S. (2008). Exercise, oxidative stress and hormesis. Ageing Res. Rev..

[31] Bo H., Kang W., Jiang N., Wang X., Zhang Y., Ji L.L. (2014). Exercise-induced neuroprotection of hippocampus in APP/PS1 transgenic mice via upregulation of mitochondrial 8-oxoguanine DNA glycosylase. Oxid. Med. Cell. Longev..

[32] Sallam N., Laher I. (2016). Exercise Modulates Oxidative Stress and Inflammation in Aging and Cardiovascular Diseases. Oxid. Med. Cell. Longev..

[33] Sahin K., Pala R., Tuzcu M., Ozdemir O., Orhan C., Sahin N., Juturu V. (2016). Curcumin prevents muscle damage by regulating NF-kappaB and Nrf2 pathways and improves performance: An in vivo model. J. Inflamm. Res..

[34] Yan Z., Zhao H., Liu A., Liu S., Zou G., Wang H. (2020). The Effects of Undenatured Type II Collagen on Inflammatory Mediators and Oxidative Stress in an Osteoarthritis Rat Model. IOP Conf. Ser. Earth Environ. Sci..

[35] Shin J.W., Seol I.C., Son C.G. (2010). Interpretation of animal dose and human equivalent dose for drug development. Korean J. Intern. Med..

[36] Feng R., Wang L., Li Z., Yang R., Liang Y., Sun Y., Yu Q., Ghartey-Kwansah G., Sun Y., Wu Y. (2019). A systematic comparison of exercise training protocols on animal models of cardiovascular capacity. Life Sci..

[37] Rivas-Estany E., Sixto-Fernandez S., Barrera-Sarduy J., Hernandez-Garcia S., Gonzalez-Guerra R., Stusser-Beltranena R. (2013). Effects of long-term exercise training on left ventricular function and remodeling in patients with anterior wall myocardial infarction. Arch. Cardiol. Mex..

[38] Wojdasiewicz P., Poniatowski L.A., Szukiewicz D. (2014). The role of inflammatory and anti-inflammatory cytokines in the pathogenesis of osteoarthritis. Mediat. Inflamm..

[39] Haseeb A., Haqqi T.M. (2013). Immunopathogenesis of osteoarthritis. Clin. Immunol..

[40] Mueller M.B., Tuan R.S. (2011). Anabolic/Catabolic balance in pathogenesis of osteoarthritis: Identifying molecular targets. PM&R.

[41] Varney J.L., Fowler J.W., Coon C.N. (2020). PSVI-33 Undenatured type II collagen mitigates inflammation and cartilage degeneration in healthy untrained Labrador retrievers after exercise. J. Anim. Sci..

[42] Bagi C.M., Berryman E.R., Teo S., Lane N.E. (2017). Oral administration of undenatured native chicken type II collagen (UC-II) diminished deterioration of articular cartilage in a rat model of osteoarthritis (OA). Osteoarthr. Cartil..

[43] Giles L.V., Koehle M.S. (2014). The health effects of exercising in air pollution. Sports Med..

[44] Powers S.K., Ji L.L., Leeuwenburgh C. (1999). Exercise training-induced alterations in skeletal muscle antioxidant capacity: A brief review. Med. Sci. Sports Exerc..

[45] Porstmann T., Santos C.R., Griffiths B., Cully M., Wu M., Leevers S., Griffiths J.R., Chung Y.L., Schulze A. (2008). SREBP activity is regulated by mTORC1 and contributes to Akt-dependent cell growth. Cell Metab..

[46] Ikeda S., Miyazaki H., Nakatani T., Kai Y., Kamei Y., Miura S., Tsuboyama-Kasaoka N., Ezaki O. (2002). Up-regulation of SREBP-1c and lipogenic genes in skeletal muscles after exercise training. Biochem. Biophys. Res. Commun..

[47] Jeong J.H., Park H.G., Lee Y.R., Lee W.L. (2015). Moderate exercise training is more effective than resveratrol supplementation for ameliorating lipid metabolic complication in skeletal muscle of high fat diet-induced obese mice. J. Exerc. Nutr. Biochem..

[48] Yu Q., Xia Z., Liong E.C., Tipoe G.L. (2019). Chronic aerobic exercise improves insulin sensitivity and modulates Nrf2 and NFkappaB/IkappaBalpha pathways in the skeletal muscle of rats fed with a high fat diet. Mol. Med. Rep..

[49] Smith I.J., Huffman K.M., Durheim M.T., Duscha B.D., Kraus W.E. (2009). Sex-specific alterations in mRNA level of key lipid metabolism enzymes in skeletal muscle of overweight and obese subjects following endurance exercise. Physiol. Genom..

[50] De Souza Cordeiro L.M., Mario E.G., Moreira C.C.L., Rodrigues A.H., Wanner S.P., Soares D.D., Botion L.M., Ferreira A.V.M. (2019). Aerobic training induces differential expression of genes involved in lipid metabolism in skeletal muscle and white adipose tissues. J. Cell Biochem..

[51] Nadeau K.J., Ehlers L.B., Aguirre L.E., Moore R.L., Jew K.N., Ortmeyer H.K., Hansen B.C., Reusch J.E., Draznin B. (2006). Exercise training and calorie restriction increase SREBP-1 expression and intramuscular triglyceride in skeletal muscle. Am. J. Physiol. Endocrinol. Metab..

[52] Larsson L., Degens H., Li M., Salviati L., Lee Y.I., Thompson W., Kirkland J.L., Sandri M. (2019). Sarcopenia: Aging-Related Loss of Muscle Mass and Function. Physiol. Rev..

[53] Silva J.M.S., Alabarse P.V.G., Teixeira V.O.N., Freitas E.C., de Oliveira F.H., Chakr R., Xavier R.M. (2018). Muscle wasting in osteoarthritis model induced by anterior cruciate ligament transection. PLoS ONE.

[54] Okada A., Ono Y., Nagatomi R., Kishimoto K.N., Itoi E. (2008). Decreased muscle atrophy F-box (MAFbx) expression in regenerating muscle after muscle-damaging exercise. Muscle Nerve.

[55] Koyama S., Hata S., Witt C.C., Ono Y., Lerche S., Ojima K., Chiba T., Doi N., Kitamura F., Tanaka K. (2008). Muscle RING-finger protein-1 (MuRF1) as a connector of muscle energy metabolism and protein synthesis. J. Mol. Biol..

[56] Mascher H., Tannerstedt J., Brink-Elfegoun T., Ekblom B., Gustafsson T., Blomstrand E. (2008). Repeated resistance exercise training induces different changes in mRNA expression of MAFbx and MuRF-1 in human skeletal muscle. Am. J. Physiol. Endocrinol. Metab..

[57] Zeng Z., Liang J., Wu L., Zhang H., Lv J., Chen N. (2020). Exercise-Induced Autophagy Suppresses Sarcopenia Through Akt/mTOR and Akt/FoxO3a Signal Pathways and AMPK-Mediated Mitochondrial Quality Control. Front. Physiol..

