# Supplementary material for: Effects of Exercise Combined with Undenatured Type II Collagen on Endurance Capacity, Antioxidant Status, Muscle Lipogenic Genes and E3 Ubiquitin Ligases in Rats
Source: Animals (Basel). 2021 Mar 17;11(3):851. doi: 10.3390/ani11030851 (PMC8002679; doi:10.3390/ani11030851)
Supplement: Supplementary file 1 [file animals-11-00851-s001.pdf]

animals

(A)

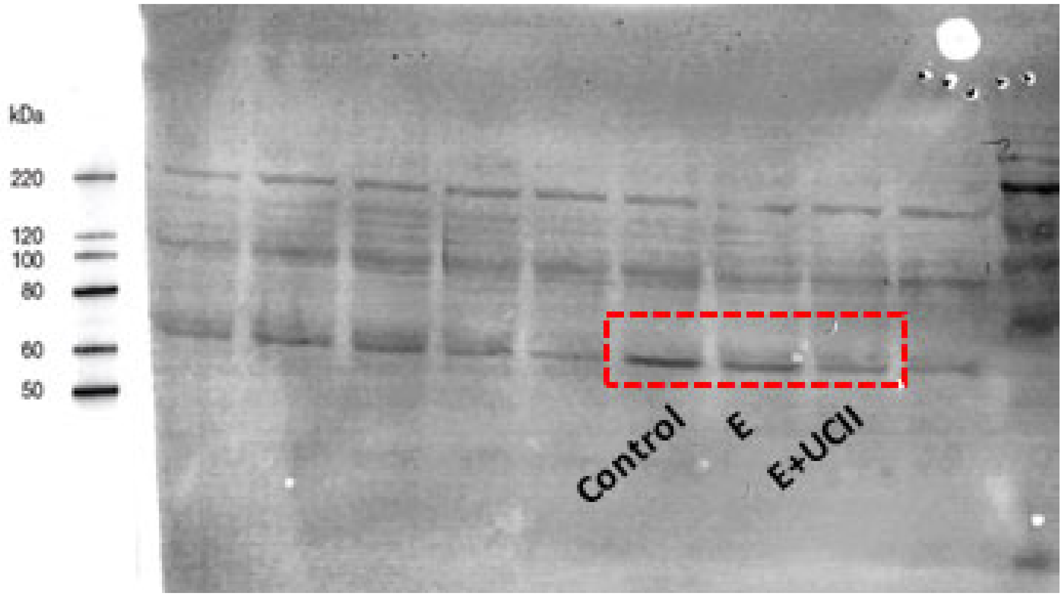
kDa
220
120
100
80
60
50
Control
E
E+UCII

(B)

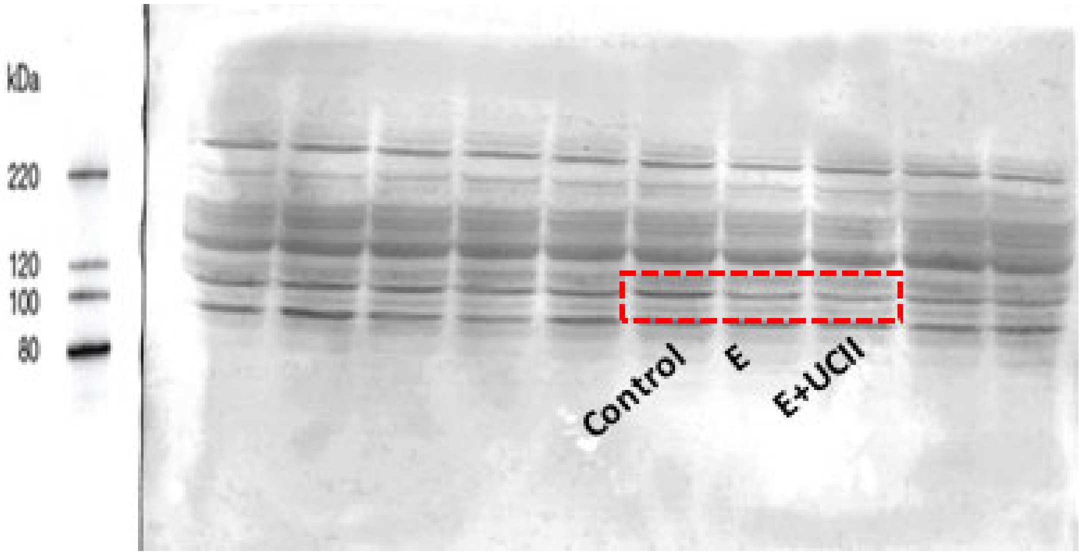
kDa
220
120
100
80
Control
E
E+UCII

(C)

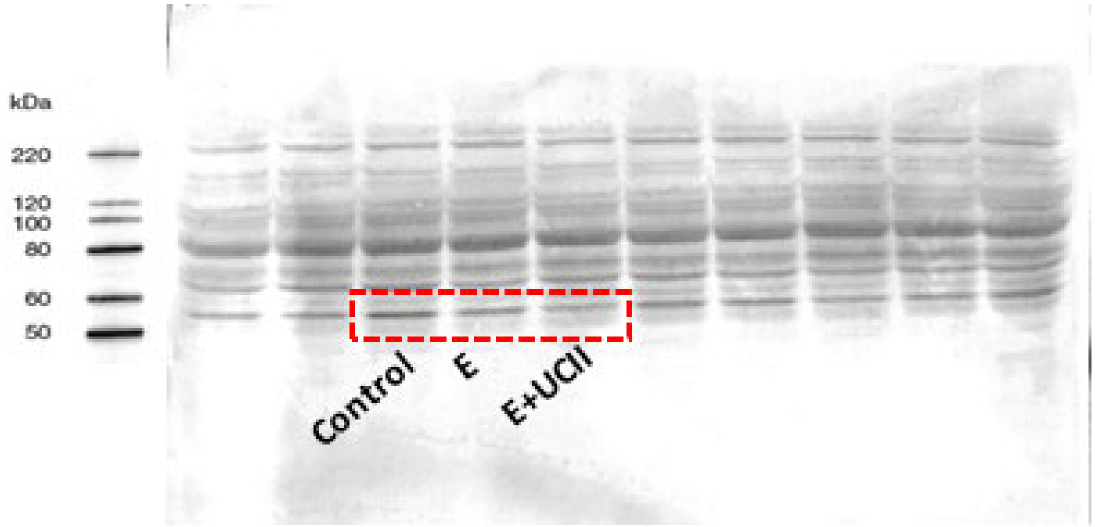
kDa
220
120
100
80
60
50
Control
E
E+UCII

(D)

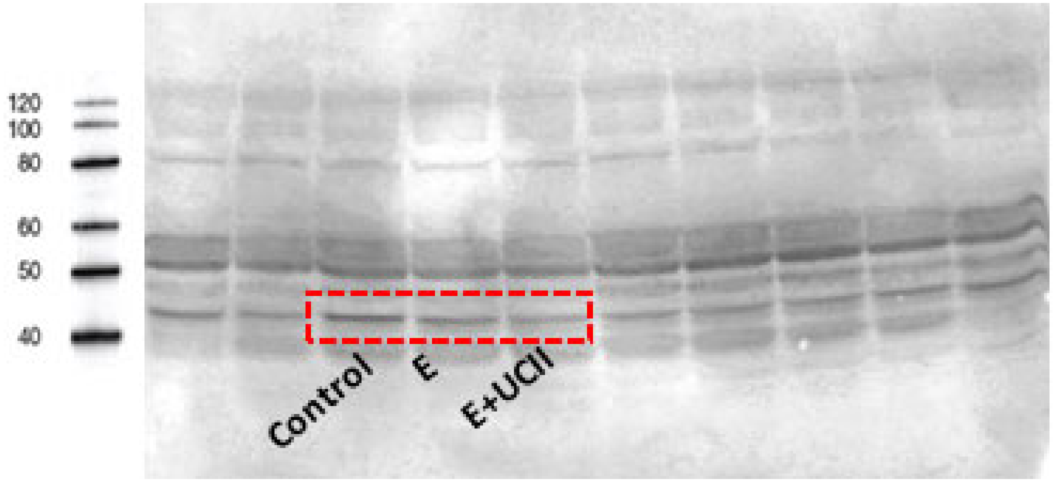
120
100
80
60
50
40
Control
E
E+UCII

(E)

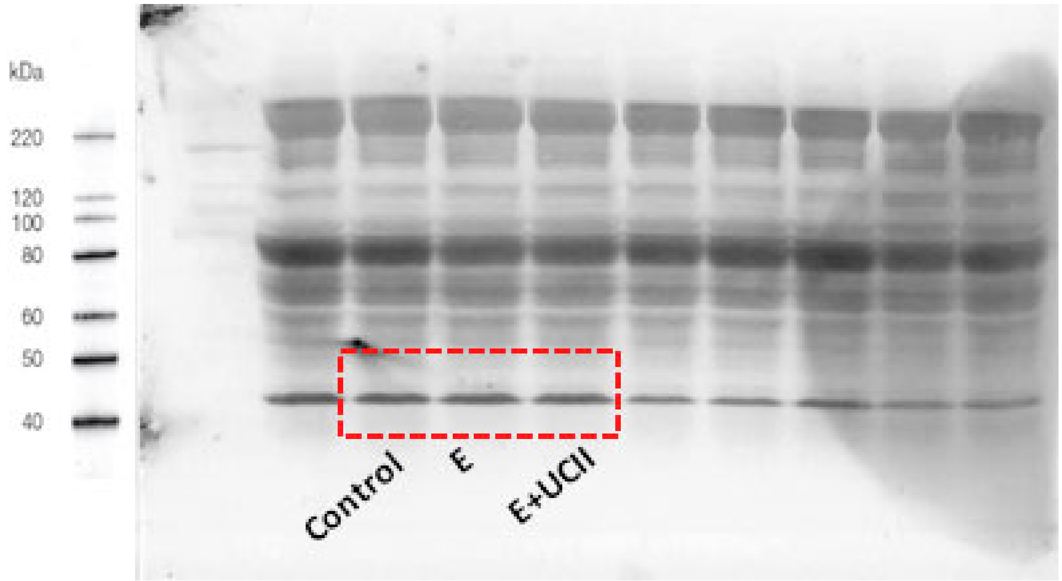

Figure S1. Full immunoblots related to Figure 4 (SREB-1C (**A**), ACLY (**B**), LXRs (**C**), FAS (**D**); β-Actin (**E**)). Each immunoblot is a representative of three independent experiments. Results shown in Figure4 are delineated by red dotted rectangles. MW (in kDa) are indicated. Control: no exercise and no UCII, E: exercised rats, Exercise + UCII, exercised rats receiving 4 mg/kg BW/day UCII.

(A)

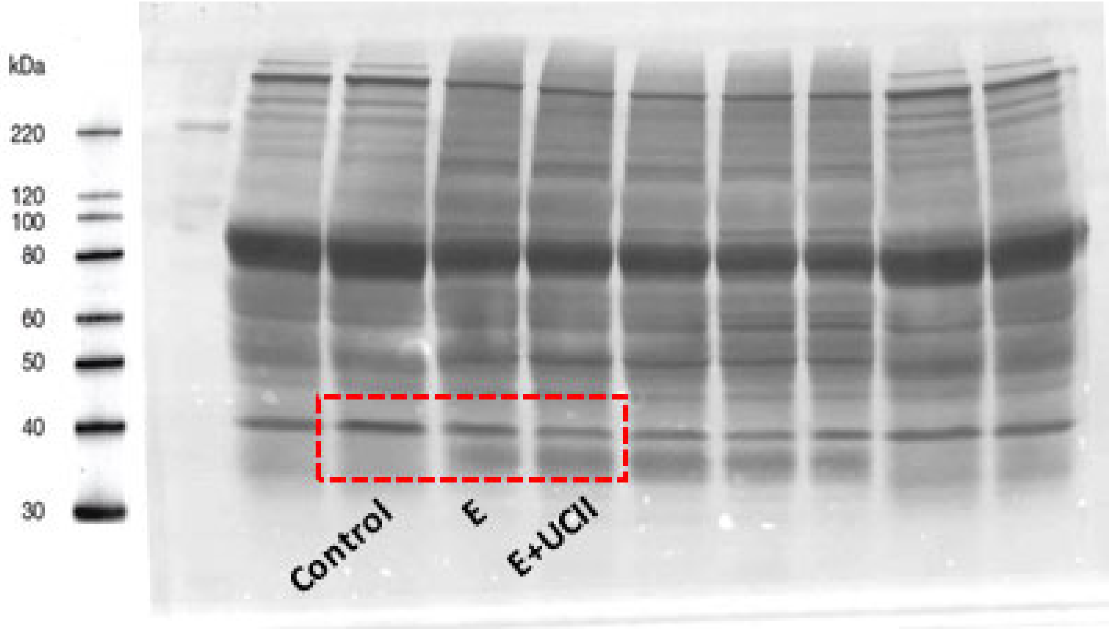
kDa
220
120
100
80
60
50
40
30
Control
E
E+UCII

(B)

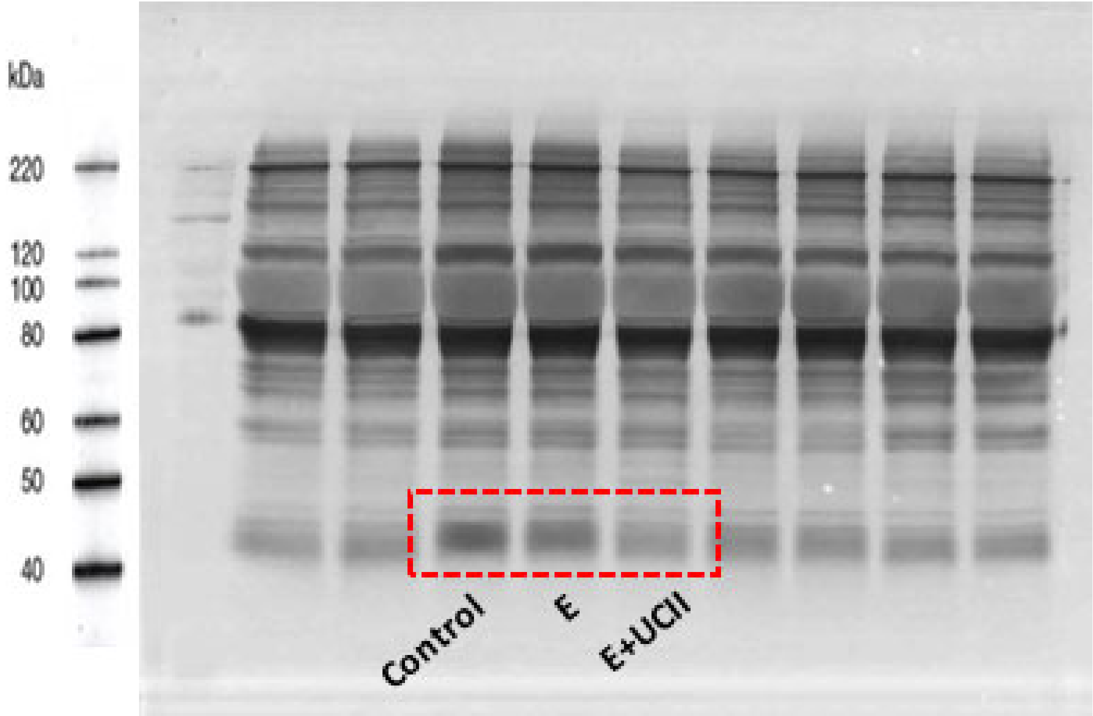
kDa
220
120
100
80
60
50
40
Control
E
E+UCII

(C)

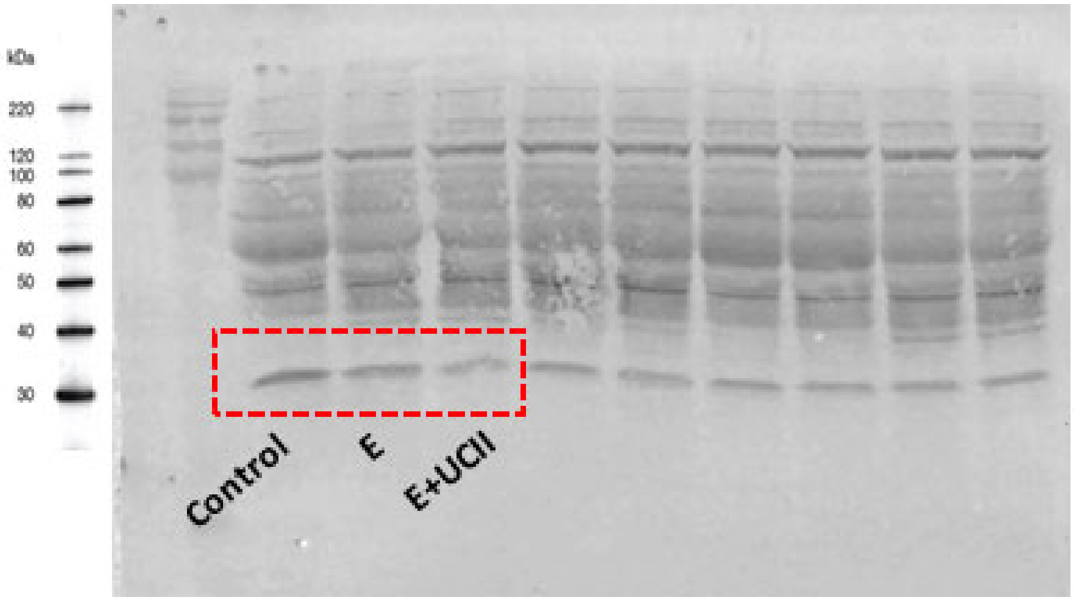
kDa
220
120
100
80
60
50
40
30
Control
E
E+UCII

(D)

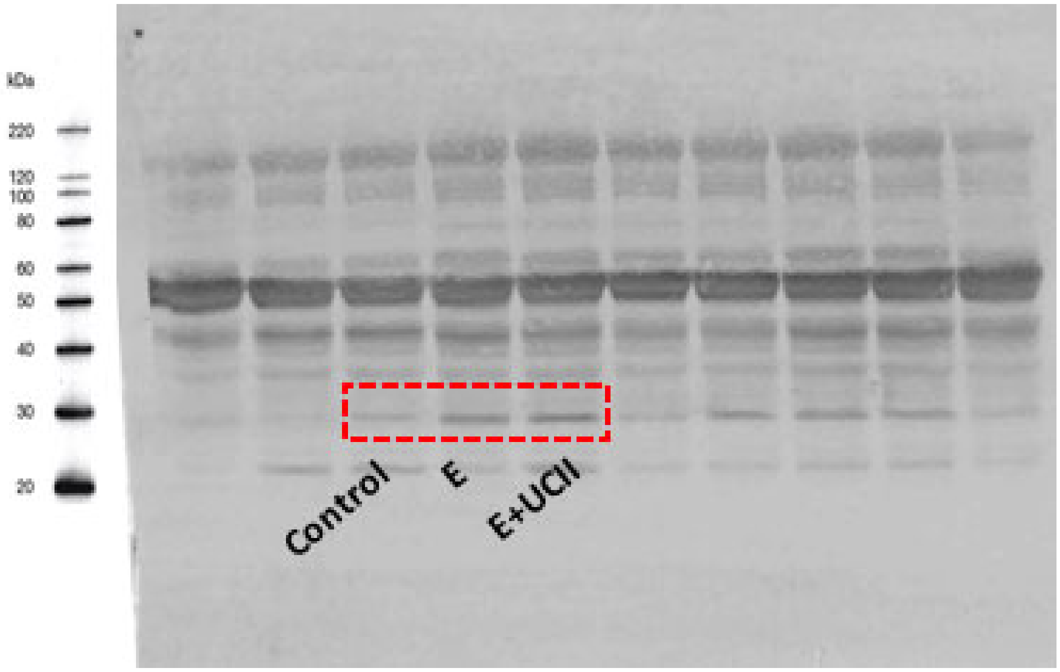
kDa
220
120
100
80
60
50
40
30
20
Control
E
E+UCII

(E)

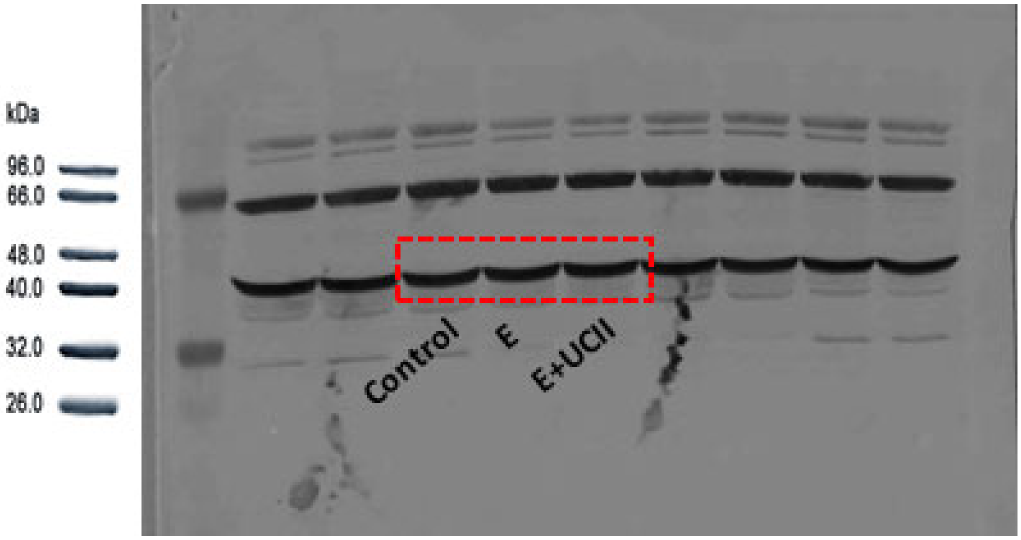

Figure S2. Full immunoblots related to Figure 5 (MAFbx (A), MuRF-1 (B), Myostatin (C), Myo D (D), and β-Actin (E)). Each immunoblot is a representative of three independent experiments. Results shown in Figure5 are delineated by rectangles. MW (in kDa) are indicated. Control: no exercise and no UCII, E: exercised rats, Exercise + UCII, exercised rats receiving 4 mg/kg BW/day UCII.

(A)

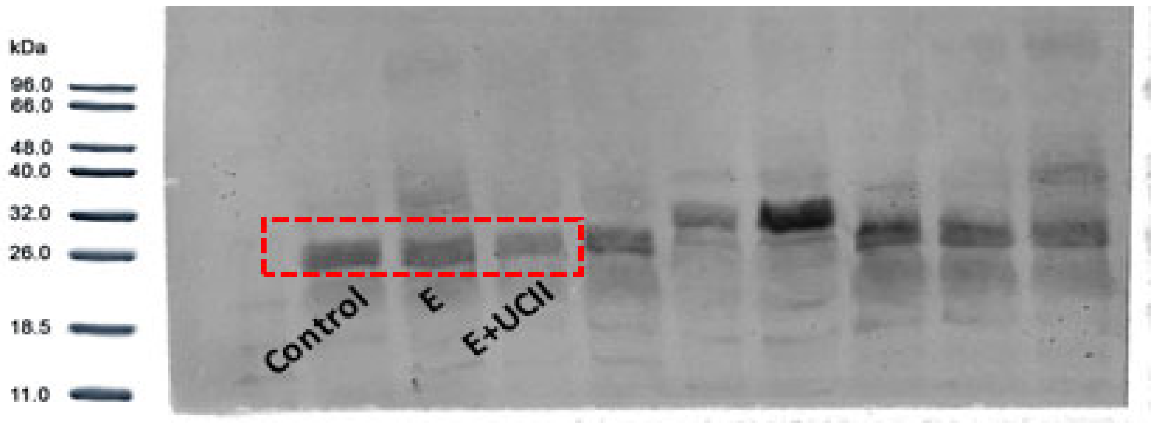
kDa
96.0
66.0
48.0
40.0
32.0
26.0
18.5
11.0
Control
E
E+UCII

(B)

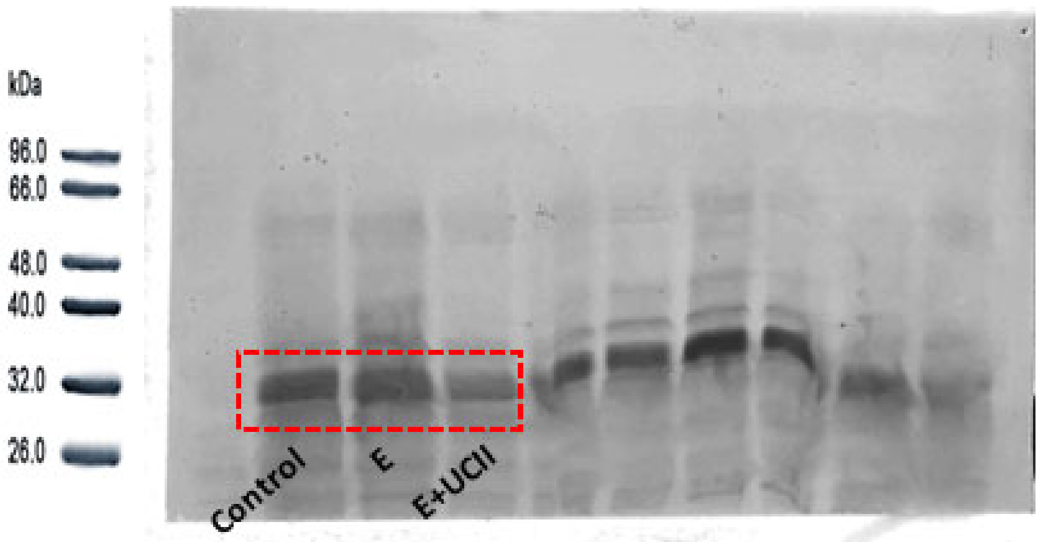
kDa
96.0
66.0
48.0
40.0
32.0
26.0
Control
E
E+UCII

(C)

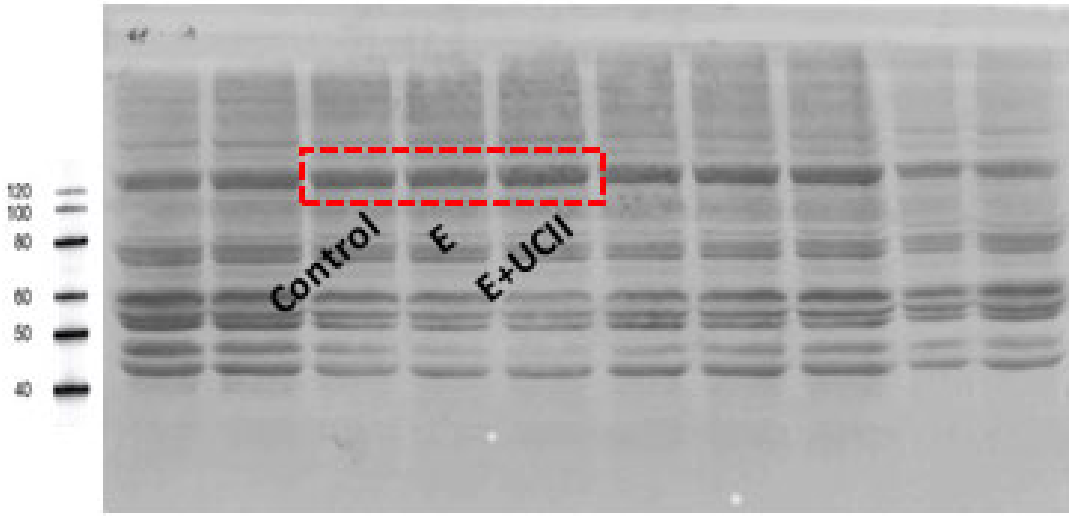

(D)

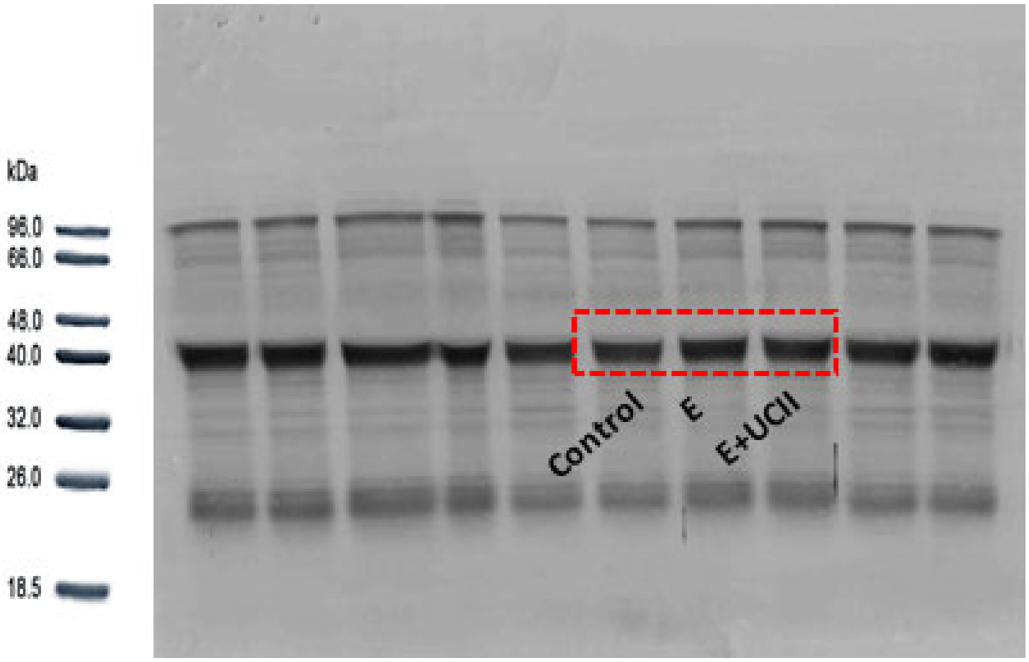

Figure S3. Full immunoblots related to Figure 6 (TNF-α (A), IL1-β (B), NCAM (C) and β-Actin (D)). Each immunoblot is a representative of three independent experiments. Results shown in Figure 6 are delineated by rectangles. MW (in kDa) are indicated. Control: no exercise and no UCII, E: exercised rats, Exercise + UCII, exercised rats receiving 4 mg/kg BW/day UCII.
